# A Distribution-Based Multiple Imputation Method for Handling Bivariate Pesticide Data with Values below the Limit of Detection

**DOI:** 10.1289/ehp.1002124

**Published:** 2010-11-19

**Authors:** Haiying Chen, Sara A. Quandt, Joseph G. Grzywacz, Thomas A. Arcury

**Affiliations:** 1 Department of Biostatistical Sciences and; 2 Department of Epidemiology and Prevention, Division of Public Health Sciences and; 3 Department of Family and Community Medicine, Wake Forest University School of Medicine, Winston-Salem, North Carolina, USA

**Keywords:** left-censoring, limit of detection, longitudinal study, maximum likelihood, multiple imputation, nondetect, repeated measures

## Abstract

**Background:**

Environmental and biomedical researchers frequently encounter laboratory data constrained by a lower limit of detection (LOD). Commonly used methods to address these left-censored data, such as simple substitution of a constant for all values < LOD, may bias parameter estimation. In contrast, multiple imputation (MI) methods yield valid and robust parameter estimates and explicit imputed values for variables that can be analyzed as outcomes or predictors.

**Objective:**

In this article we expand distribution-based MI methods for left-censored data to a bivariate setting, specifically, a longitudinal study with biological measures at two points in time.

**Methods:**

We have presented the likelihood function for a bivariate normal distribution taking into account values < LOD as well as missing data assumed missing at random, and we use the estimated distributional parameters to impute values < LOD and to generate multiple plausible data sets for analysis by standard statistical methods. We conducted a simulation study to evaluate the sampling properties of the estimators, and we illustrate a practical application using data from the Community Participatory Approach to Measuring Farmworker Pesticide Exposure (PACE3) study to estimate associations between urinary acephate (APE) concentrations (indicating pesticide exposure) at two points in time and self-reported symptoms.

**Results:**

Simulation study results demonstrated that imputed and observed values together were consistent with the assumed and estimated underlying distribution. Our analysis of PACE3 data using MI to impute APE values < LOD showed that urinary APE concentration was significantly associated with potential pesticide poisoning symptoms. Results based on simple substitution methods were substantially different from those based on the MI method.

**Conclusions:**

The distribution-based MI method is a valid and feasible approach to analyze bivariate data with values < LOD, especially when explicit values for the nondetections are needed. We recommend the use of this approach in environmental and biomedical research.

Analytic procedures for environmental and biomedical data often have a limit of detection (LOD), which is defined as the lowest concentration level of a substance that can be determined to be statistically different from a blank value with a stated confidence level. Because values < LOD (nondetections) cannot be determined precisely, data are missing for the lower end of the distribution (i.e., left- censored). However, values < LOD are informative because they indicate that the analyte has a concentration between 0 and LOD, and simply excluding such values from analyses may substantially bias results (Hornung and Reed 1990). A variety of methods have been proposed for handling values < LOD, as described in detail by Helsel (2005b, 2010). For example, simple substitution methods, parametric methods, nonparametric Kaplan-Meier methods, and robust regression on order statistics methods can be used to obtain summary statistics (e.g., means, standard deviations, medians, and percentiles) for left-censored data. Simple substitution methods, parametric methods based on survival techniques, and nonparametric methods, such as the Wilcoxon rank-sum test, can be used for group comparisons. For example, Millard and Deverel (1988) used nonparametric methods to compare zinc concentrations in shallow groundwater collected from two different locations.

For more in-depth analysis such as regression modeling, the simple substitution methods are the easiest to implement. These methods involve substituting a single value chosen from the interval from zero to the LOD for each value < LOD. The most commonly used substitutions are zero, LOD/2,
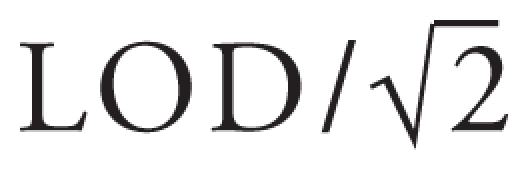
, or LOD (Barr et al. 2006). However, replacing a sizable portion of the data with a single value increases the likelihood that the resulting parameter estimates will be biased (Helsel 1990). Consequently, standardized data quality assessment guidelines outlined by the U.S. Environmental Protection Agency (EPA) do not recommend simple substitution when 15% or more of values are < LOD (U.S. EPA 2000).

Instead of simple substitution for the values < LOD, an alternative is to assume a specific parametric distribution (e.g., left-censored log-normal distribution) for the left-censored data. Likelihood-based estimation can be performed based on the detected values and the observed percentage of values < LOD. These distributional methods have been applied to both cross-sectional data (Lynn 2001; Taylor et al. 2001) and longitudinal data (Hughes 1999; Jacqmin-Gadda et al. 2000; Lyles et al. 2001a, 2001b; Thiébaut and Jacqmin-Gadda 2004) when the analyte is the outcome of interest. However, they do not perform well in the situations where the assumed parametric distribution is incorrect, the data set is small, and/or the percentage of censoring is high (Helsel 2005a). In addition, these pure parametric approaches are not applicable when the analyte is an independent variable (exposure or predictor) rather than the outcome.

Distribution-based multiple imputation (MI) methods offer an increasingly compelling alternative for the analysis of left-censored data (Baccarelli et al. 2005; Huybrechts et al. 2002; Lubin et al. 2004). These methods use maximum likelihood estimates (MLEs) to estimate distribution parameters based on the available data (both the observed values > LOD and the proportion of values < LOD) that are subsequently used to impute values for observations < LOD so that a complete data set is created. Because the imputed values cannot be treated as actual measured data, the imputation process is usually repeated several times to create multiple complete data sets. Each complete data set is analyzed, and the results are combined to account for the uncertainty resulting from MI methods (Little and Rubin 2002). Distribution-based MI methods assume that the observations > and < LOD come from a common parametric distribution. They are robust to mild or moderate departures of the observed data from the assumed underlying distribution (Huybrechts et al. 2002), and they provide accurate estimates of population parameters for moderate sample size (at least 50 observations) even when the proportion of nondetects is high (Baccarelli et al. 2005; Lubin et al. 2004). In addition, they can be applied when the analyte of interest is an outcome or a predictor.

Left-censored longitudinal data pose analytical challenges to the application of the distribution-based MI methods. For cross-sectional data, only the mean and the variance of a specified univariate distribution need to be estimated. However, in the longitudinal setting, the mean vector and the entire variance–covariance matrix must be estimated so that MI can be performed. The objective of this article is to illustrate how left-censored bivariate data (i.e., longitudinal data with observations < LOD for an analyte measured on two different occasions, or cross-sectional data with observations < LOD for two different analytes) can be imputed based on a bivariate normal distribution and analyzed using an MI approach. We first derive the likelihood function for a truncated bivariate normal distribution with missing data then describe an MI method for values < LOD. Next we present results of a simulation study to evaluate the ML and MI estimators. Finally, we illustrate the application of the distribution-based MI method using data from the Community Participatory Approach to Measuring Farmworker Pesticide Exposure (PACE3) study.

## Methods

### Estimating the parameters from a bivariate normal distribution

Let (*x_i_*, *y_i_*) denote two measures on subject *i*, *i* = 1, . . ., *n*. In practice, (*x_i_*, *y_i_*) can be repeated measures of the same analyte (as in a longitudinal analysis) or measures of two different analytes. We assume that (*x_i_*, *y_i_*) are independently and identically distributed as bivariate normal with mean (μ*_x_*, μ*_y_*), variance (σ^2^*_x_*,σ^2^*_y_*), and correlation coefficient ρ. It follows that the marginal distributions and the conditional distributions are also normal. We further assume that both *x_i_* and *y_i_* are subject to left censoring. For simplicity, we use the same known LOD *L* for both *x_i_* and *y_i_* in the derivation below, but differences in the LODs for *x_i_* and *y_i_* (e.g., because of differences in laboratory procedures) can be incorporated with a slight modification of the likelihood function. In addition to data that are missing because of values < LOD (not missing at random), we also may have missing data for *x_i_* and *y_i_* for other reasons (e.g., because an analytic sample was not obtained), and we assume in this article that such data are missing at random (MAR). Therefore, the likelihood function depends on eight possible data patterns (*l*_1_ – *l*_8_) determined by three possible types of values (observed, < LOD, or MAR) for the two variables, *x_i_* and *y_i_* (Lyles et al. 2001b).

When both (*x_i_*, *y_i_*) are known (> LOD), their contribution to the likelihood function (*l*_1_) is simply the joint density function of a bivariate normal distribution. That is,


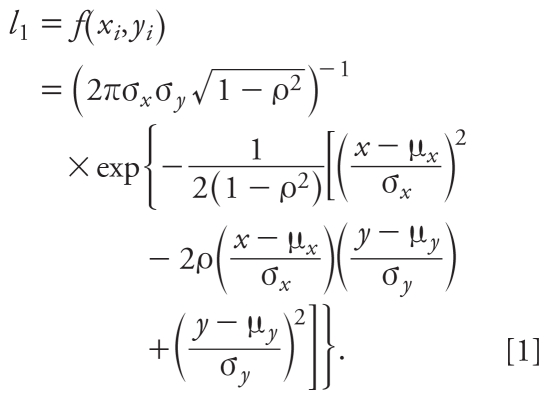


When *x_i_* is known and *y_i_* is < LOD, their contribution to the likelihood function (*l*_2_) can be expressed as the product of the marginal distribution of *x_i_* and the conditional probability of *y_i_* < LOD given that *x_i_* is observed:


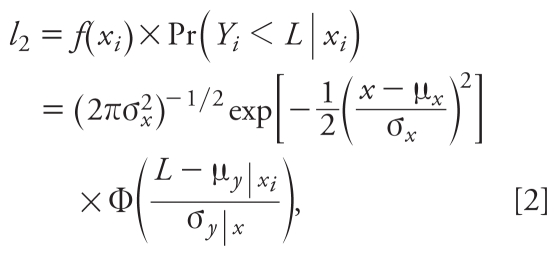


where μ*_y|xi_* = μ*_y_* + ρ(σ*_y_* */*σ*_x_*)(*x_i_* − μ*_x_*), σ^2^*_y|x_* = σ^2^*_y_* (1 − ρ^2^), and Φ represents the cumulative distribution function of a standard normal. Similarly, when *y_i_* is known and *x_i_* is < LOD, their contribution to the likelihood function (*l*_3_) can be expressed as


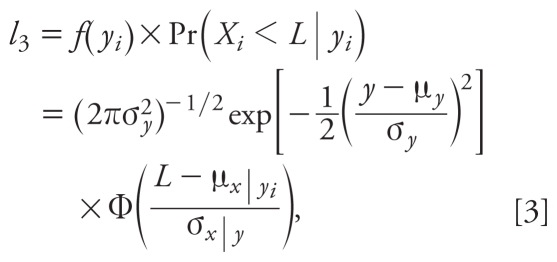


where μ*_x|yi_* = μ*_x_* *+* ρ(σ*_x_* */*σ*_y_*)(*y_i_* − μ*_y_*) and σ^2^*_x|y_* = σ^2^*_x_* (1 − ρ^2^). When both *x_i_* and *y_i_* are < LOD, their contribution to the likelihood function (*l*_4_) is the probability of *x_i_* and *y_i_* both being < *L* (the value of the LOD) under a bivariate normal distribution:


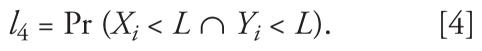


This can be derived directly from *f*(*x_i_*, *y_i_*) and evaluated through a close numerical approximation.

When *x_i_* is known and *y_i_* is MAR, their contribution to the likelihood function (*l*_5_) is simply the marginal distribution function of *x_i_*. Similarly, when *y_i_* is known and *x_i_* is MAR, their contribution to the likelihood function (*l*_6_) is the marginal distribution function of *y_i_*. When *x_i_* is < LOD and *y_i_* is MAR, or when *y_i_* is < LOD and *x_i_* is MAR, their contributions to the likelihood function *l*_7_ and *l*_8_ are the unconditional probability of *x_i_* < LOD and *y_i_* < LOD, respectively:


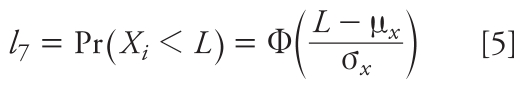



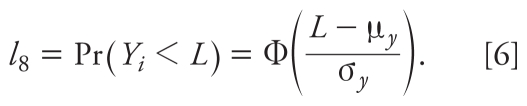


The final likelihood function is the product of *l*_1_ through *l*_8_ over the entire sample space. The log-likelihood function can then be maximized using various optimization routines available in many commercial software packages. In this article, we used a nonlinear optimization routine by Newton-Raphson ridge method in SAS IML (SAS Institute Inc., Cary, NC).

In our article, we use bivariate normal distributions as the basis of our studies, but in some circumstances observations < LOD may have some clusters of true zero values, and in these cases, imputing a strictly positive value between 0 and LOD (or a value below logarithmic LOD) will bias the results. Using a mixture distribution such as a zero-inflated lognormal to define the likelihood function is a feasible method to address this issue but is beyond the scope of this article.

### MI for values < LOD

After the log-likelihood function is created using all available data [including observations with known values > LOD (detections), observations with values < LOD (nondetections), and observations that are MAR], we can derive MLEs of (μ*_x_*, μ*_y_*), (σ^2^*_x_*, σ^2^*_y_* ), and ρ. Let (μ̂*_x_*, μ̂*_y_*), (σ̂^2^*_x_*, σ̂^2^*_y_*), and ρ̂ be the corresponding MLEs of parameters for the bivariate normal distribution of *X* and *Y*. The parameter estimates for a conditional distribution such as μ̂*_y_*_|_*_xi_* and σ̂^2^*_y|x_* can be calculated based on standard bivariate normal theory and the invariance property of MLE. Although values < LOD can be imputed by sampling from the estimated distribution based on (μ̂*_x_*, μ̂*_y_*), (σ̂^2^*_x_*, σ̂^2^*_y_*), and ρ̂, we note that the MLEs are themselves estimated with uncertainty. Therefore, to account for uncertainty in parameter estimation, we use estimates from a series of bootstrapped samples based on maximum likelihood approach to impute values < LOD (Little and Rubin 2002). Bootstrap data are generated by random sampling with replacement (Efron 1979) so that each bootstrap sample is the same size as the original sample (including detections, nondetections, and MAR observations). For each bootstrap data set, the likelihood function is constructed as described above to obtain estimates (μ̃*_x_*, μ̃*_y_*), (σ̃^2^*_x_*, σ̃^2^*_y_*), and ρ̃. Because each bootstrap data set yields different estimates for (μ*_x_*, μ*_y_*), (σ^2^*_x_*, σ^2^*_y_*), and ρ, we have a series of (μ̃*_x_*, μ̃*_y_*), (σ̃^2^*_x_*, σ̃^2^*_y_*), and ρ̃ to use for subsequent imputations, thus accounting for the uncertainty in the parameter estimation. Then, one imputation is carried out for nondetections in the original data set using one set of (μ̃*_x_*, μ̃*_y_*), (σ̃^2^*_x_*, σ̃^2^*_y_*), and ρ̃ as follows.

When *x_i_* is known and *y_i_* is < LOD, a random draw from the conditional distribution of *y_i_* given the observed value of *x_i_* truncated at the LOD is used to impute a value for *y_i_*. In this way, we ensure that only values < LOD are imputed for nondetections. Similarly, a value of *x_i_* can be imputed when *y_i_* is known and *x_i_* is < LOD. In the situation where both *x_i_* and *y_i_* are < LOD, both values are imputed simultaneously from a truncated bivariate normal distribution with parameters (μ̃*_x_*, μ̃*_y_*), (σ̃^2^*_x_*, σ̃^2^*_y_*), and ρ̃. When either *x_i_* or *y_i_* is MAR and the other variable is < LOD, the < LOD value is imputed based on the estimated marginal distribution (a truncated univariate normal).

The whole process, that is generating a bootstrap sample, estimating (μ*_x_*, μ*_y_*), (σ^2^*_x_*, σ^2^*_y_*) and ρ for the bootstrap sample using maximum likelihood and imputing data that are < LOD based on (μ̃*_x_*, μ̃*_y_*), (σ̃^2^*_x_*, σ̃^2^*_y_*), and ρ̃ are repeated to create multiple imputed data sets, thereby accounting for the uncertainty in the imputed values. It has been shown that the efficiency of an estimate based on *m* imputed data sets is approximately (1 + γ/*m*)^−1^, where γ is the rate of missing information for the quantity being estimated (Little and Rubin 2002). Unless γ is very high (e.g., 80–90%), good efficiencies can generally be achieved with 3–10 imputed data sets. Thus, we used five bootstrap samples to obtain five sets of distribution parameter estimates, from which we generated five imputed data sets on the original data in this analysis. Because the imputed nondetectable values are all random draws based on the estimated bivariate normal distribution, the correlation between the repeated measures in a longitudinal study is retained, even with observations < LOD.

### Simulation study

**Figure 1:**
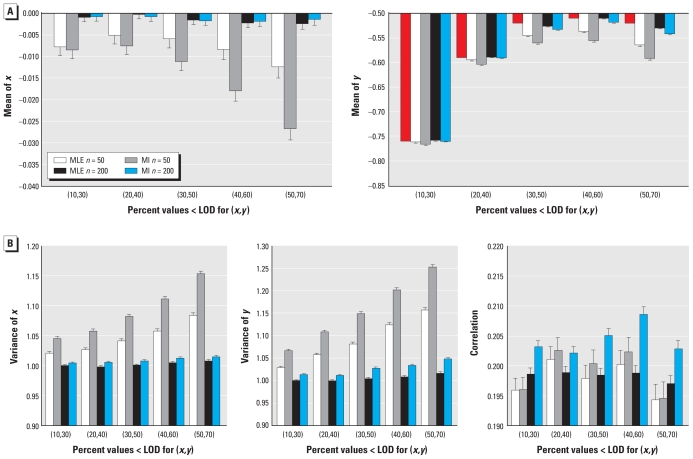
(*A*) MLEs and MI estimates for μ*_x_* (left) and μ*_y_* (right) from 5,000 simulated samples. The true value for μ*_x_* is 0; the true values for μ*_y_* are −0.76, −0.59, −0.52, −0.51, and −0.52 for (10, 30), (20, 40), (30, 50), (40, 60), and (50, 70) percent of (*X*, *Y*) < LOD, respectively. The true value of μ*_y_* is represented by the red reference bars. (*B*) MLEs and MI estimates for σ^2^*_x_* (left), σ^2^*_y_* (middle), and ρ (right) from 5,000 simulated samples. The true values for σ^2^*_x_*, σ^2^*_y_*, and ρ are 1, 1, and 0.2, respectively.

We conducted a simulation study to evaluate the sampling property of MLEs and MI estimators under different scenarios. For each scenario, we calculate MLEs for the distribution parameters from the (simulated) original data set. In addition, we estimate distribution parameters for each of five bootstrap samples, use the estimated parameters to impute values < LOD for the original sample from which we generated the five bootstrap samples, and combine results across the five imputed data sets to obtain MI estimates. For all scenarios, we assume that the bivariate normal random variables (*X*, *Y*) have population parameters μ*_x_* = 0, σ^2^*_x_* = σ^2^*_y_* = 1. We varied the correlation between *X* and *Y* such that ρ = 0.2, 0.5, or 0.8, and we set the value of μ*_y_* and the proportion of observations < LOD so that the marginal distributions of *X* and *Y* were subject to various degrees of left censoring (for example, μ*_y_* = −0.76 with 10% of observations < LOD for *X* and 30% < LOD for *Y*; for details, see Figure 1). Finally, we evaluated the performance of these methods for two different sample sizes (*n* = 50 and *n* = 200). For each combination of percentage of censoring, correlation coefficient, and sample size, we generated 5,000 replicates to approximate the sampling distribution of the MLE and MI estimator.

The overall pattern was very similar for different correlations (details not shown). Therefore, to simplify the presentation of results, we report results of scenarios where ρ = 0.2 only. Figure 1 shows the MLEs and MI estimates for (μ*_x_*, μ*_y_*), (σ^2^*_x_*, σ^2^*_y_*), and ρ from each simulation. The error bars represent the standard error (SE) of each estimate. As expected, MLE shows minimal bias when the sample size is large (*n* = 200), although estimates are slightly biased when the proportions of observations < LOD are large (50–70%). MLEs are more biased when the sample size is small (*n* = 50), particularly as proportions of censored observations (< LOD) increase. For example, when 50% of *X* and 70% of *Y* are < LOD, MLEs overestimate σ^2^*_x_* and σ^2^*_y_* by 8% and 16%, respectively, with a sample size of 50. Overall, the MI estimates are fairly comparable to the MLEs when the sample size is large or the degree of censoring is low. However, the MI estimates tend to be more biased than the MLEs when the sample size is small and there is a large amount of censoring (e.g., σ^2^*_x_* and σ^2^*_y_* are overestimated by 15% and 25%, respectively, when *n* = 50 and 50% of *X* and 70% of *Y* are < LOD). Finally, the MI estimates are slightly more variable than MLEs as indicated by larger SEs. This is probably due to the bootstrapping and MI process.

### Motivating example

We consider data from the Community Participatory Approach to Measuring Farmworker Pesticide Exposure (PACE3) study. This is a longitudinal study examining multiple pathways of farmworker pesticide exposure, including work environment, home environment, work and household behaviors, and community factors. A total of 287 farmworkers from 11 counties in eastern North Carolina were included in the study in 2007. Detailed information concerning the design and sample collection for the PACE3 study can be found in Arcury et al. (2009).

For this analysis we focus on the concentrations of the urinary acephate (APE). APE is an organophosphorus (OP) pesticide widely used to treat tobacco (Southern and Sorenson 2008). As with all OP insecticides, APE is a neurotoxin. The immediate health effects of small doses of OP insecticides can include nausea and vomiting, burning of the nose or throat, red or burning eyes, rash, dizziness, headache, blurred vision, and muscle weakness (Reigart and Roberts 1999; Sanborn et al. 2004). Immediate health effects of a large dose of OP insecticides can be severe and include loss of consciousness, coma, and death. Long-term health effects of exposure to OP insecticides such as APE can occur, particularly when exposures are repeated, including increased risk of neurological decline in adults, impaired neurobehavioral development of children, several cancers, and reproductive health problems (Eskenazi et al. 2007; Perry et al. 2007; Weichenthal et al. 2010).

In this longitudinal study, urinary pesticide concentrations were measured across four periods in the agricultural season (period 1, May 1 to June 8; period 2, June 9 to July 7; period 3, July 8 to August 5; period 4, August 6 to September 4). Farmworkers involved in activities such as topping, harvesting, or curing pesticide-treated tobacco were almost definitely exposed to APE. Therefore, we limited our analyses to the repeated measures from periods 3 and 4 when most of these activities occurred. In addition, we excluded observations from farmworkers with an APE measurement < LOD who did not top, harvest, or barn tobacco during the corresponding period, to avoid imputing a positive value for a true zero. The final sample comprised 209 farmworkers.

In each period, the farmworkers also responded to interviewer-administered questionnaires to assess immediate symptoms (nausea, burning nose or throat, rash, vomiting, dizziness, headache, red or burning eyes, blurred vision, and/or weak or heavy arms in the last 3 days) related to potential pesticide poisoning. Interviews were completed with individual farmworkers at about 1-month intervals. Having any of the nine symptoms (yes/no) was our primary health outcome in this analysis.

## Data Analysis and Results

Preliminary analyses indicated that the log-normal distribution is a reasonable assumption for APE concentrations. We then log-transformed all APE values > LOD and the LOD itself so that we could apply the method described above to estimate distributional parameters of a bivariate normal. Table 1 summarizes the eight different data patterns that contribute to the overall likelihood function. For the two repeated APE measures in periods 3 and 4, about 38% of observations had both values > LOD, and about 8% had both values < LOD. Overall, about 13% and 39% of the data were < LOD in periods 3 and 4, respectively. MLEs of the mean and variance of log(APE) concentrations were −0.41 and 4.75, respectively, for period 3, and −2.70 and 11.14 for period 4. Estimated APE concentrations in period 4 were lower and more variable than in period 3, but, as expected, measurements from these two periods were positively correlated (ρ = 0.20).

**Figure 2:**
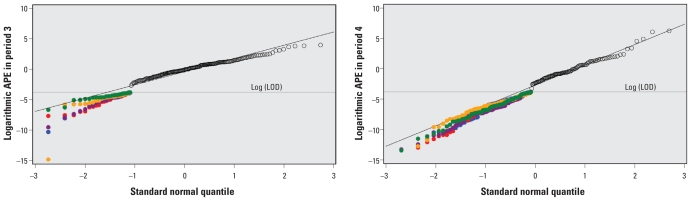
Normal Q-Q plots for logarithmic APE in periods 3 and 4 normal Q-Q plots of the observed log(APE) values [> log(LOD)] and the imputed APE values [< log(LOD)] from each imputed data set for period 3 (*A*) and period 4 (*B*). The observed values > log(LOD) [open circles, above the log(LOD) reference line] are identical for all five data sets. Imputed values < log(LOD) differ between the data sets (indicated by different-colored dots). Diagonal reference lines indicate the estimated bivariate normal distribution based on MLEs for each period. For simplicity, reference lines for the estimated distributions from the five imputed data sets are not shown.

The MLEs themselves are estimates. Therefore, we created five bootstrap data sets and obtained five sets of distributional parameter estimates that we used to impute values < LOD for the original sample. We used normal quantile–quantile (Q-Q) plots to examine the overall distribution of the observed (> LOD) and the imputed (< LOD) log(APE) concentrations for the five imputed data sets. A Q-Q plot compares the empirical quantiles based on the data with the quantiles from a standard normal distribution. Usually a diagonal reference line is drawn with estimated population mean as intercept and estimated population standard deviation as slope. If the points on a Q-Q plot fall on this straight reference line, it is supportive of a normal distribution assumption. Figure 2 shows the normal Q-Q plots of log(APE) concentrations. We present the five imputed data sets together in one panel to describe the variability of the imputed values across different imputations. For simplicity, diagonal reference lines for the estimated distributions from the five imputed data sets are not shown. Instead, the diagonal reference lines in Figure 2 are based on the MLEs. In addition, we drew a horizontal reference line valued at log(LOD) to indicate that all the data points above this line were observed (> LOD) and therefore common to all the imputed data sets and all the data points below this line (< LOD) were imputed and vary from imputation to imputation. Figure 2 indicates that the imputed values and the observed values > LOD in general conform to the estimated bivariate normal distribution. However, there is a slight curvature around the LOD, especially in period 3, that may reflect a lack of data between the LOD (0.023) and the minimum measured value > LOD in period 3 (0.07). Figure 2 also shows that the imputed data points overlapped substantially.

We then performed linear regression on each imputed data set with APE as the outcome and combined results from the five imputed data sets using SAS PROC MI ANALYZE to account for between- and within-imputation variability. We applied a linear mixed effects model to test whether the mean APE concentration in period 3 was significantly different from that in period 4. The estimated mean log(APE) ± SE was −0.48 ± 0.23 log ng/mL for period 3 and −3.09 ± 0.26 log ng/mL for period 4 (*p* < 0.0001). The model can then be expanded easily to incorporate more explanatory variables and assess their associations with outcome (APE).

Next we used logistic regression models to estimate the association between APE exposures and the presence of any symptoms of potential pesticide poisoning, using a generalized estimating equation approach to account for the correlations of longitudinal data. Overall, 42% and 45% of farmworkers experienced at least one of the nine symptoms in periods 3 and 4, respectively. We found no significant log(APE) by time interaction (*p* = 0.15), so our main effect model included only time and log(APE) as explanatory variables. Time was not significantly associated with the outcome (*p* = 0.17), but a unit increase in log(APE) was significantly associated with the likelihood of reporting any symptoms (odds ratio = 1.07; 95% confidence interval, 1.00–1.14).

Finally, to illustrate empirically the benefit of this distribution-based MI method, we repeated the analyses above with the observations < LOD excluded completely or replaced by log(LOD)/2 or log(LOD). The overall results from these ad hoc methods were substantially different from those based on the MI method (Table 2), resulting in markedly higher estimates for the mean logarithmic APE concentrations in both periods and for the association between APE concentrations and the presence of symptoms.

## Discussion

Left-censored data are common in environmental and biomedical research when laboratory analyses of substances of interest are constrained by a lower LOD. Additionally, researchers often need the explicit values for the measurements < LOD to test scientific hypotheses. For example, one needs to quantify the association between semen concentrations and pesticide concentrations in reproductive research or the association between HIV RNA concentrations and age at seroconversion in AIDS research. Without valid statistical methods to fill in the values < LOD, researchers frequently have to resort to categorizing the left-censored data in analyses, which may lead to bias and substantial loss of efficiency (Taylor and Yu 2002). Therefore, the development of a valid imputation method is critical for environmental and biomedical research.

Single substitution methods are not recommended unless the proportion of values < LOD is small, usually < 10% (Helsel 2005b). MI methods are robust to mild or moderate deviations of observed data from assumed underlying distribution and take into account the uncertainty due to imputation. In this article we expand distribution-based MI methods for left-censored data from a cross-sectional setting (Lubin et al. 2004) to a longitudinal setting. In our PACE3 study, this imputation method allows an examination of the associations of pesticide exposure with immediate health outcomes measured over time. This approach allows us to see that even at low concentrations, APE exposure, as indicated by measures of the urinary metabolite APE, increases the risk of symptoms known to result from exposure to OP pesticides. Such analyses have not been available with the simple substitution approach when the number of values < LOD has been large or with the MLE approach because the left-censored data are used as a predictor.

Literature has shown that MLE does not perform well when the degree of left censoring is as large as 80% (Helsel 2005b). In fact, it is recommended that only the percentage of nondetections be reported under such heavy censoring. Therefore, because MLE serves as the basis of the distribution-based MI method, we did not examine scenarios in which > 80% of data were < LOD. Overall, our simulation results demonstrated that the MLEs based on the available information provide a solid theoretical basis for generating explicit values for the measurements < LOD. The MLEs were consistent estimators of true parameter values as expected. Simulation results also showed that the imputed values and the observed measurable values together yielded consistent estimates for the assumed underlying distribution that were not substantially influenced by the extent of correlation between the two measurements. Thus, the distribution-based MI method is valid and feasible for handling longitudinal left-censored data, and we therefore encourage its application in environmental and biomedical research.

We used the normal distribution as the basis for imputing values < LOD. This is often a reasonable assumption because a large number of the environmental exposures and biomarkers follow a normal distribution after log transformation. However, there are situations where data may actually come from other parametric distributions. For example, gamma distributions have many similarities with lognormal distributions, and the two can be mistaken for one another. When the distributional assumption is severely violated, some distribution-free imputation methods may be considered. Schisterman et al. (2006) proposed using least squares methods in a regression setting to find a constant that can be imputed for all the values < LOD while keeping the estimated regression coefficients unbiased. The application of this type of nonparametric imputation approach in a longitudinal setting needs to be studied further. Overall, a check of the distribution assumption is highly recommended before one proceeds with more complex analyses.

Finally, we note that the principle of the distribution-based MI method can be extended to more than two repeated measures. However, as the number of repeated measures increases, the number of potential data patterns increases. This leads to a more complicated derivation of the likelihood function. Meanwhile, the number of distribution parameters that need to be estimated increases accordingly. In particular, the complexity of the assumed variance covariance structure can pose analytical challenges to the optimization techniques. On the other hand, an overly simplistic variance covariance structure may lead to bias in the estimation of MLEs and have a subsequent negative effect on the imputation process. More research is needed in this area.

## Figures and Tables

**Table 1:** Different data patterns for deriving maximum likelihood function (frequencies and percentages).

	Period 3		
Period 4	> LOD	< LOD	Missing
> LOD	80 (38.3%)	7 (3.3%)	6 (2.9%)
< LOD	63 (30.1%)	16 (7.7%)	3 (1.4%)
Missing	29 (13.9%)	5 (2.4%)	—

**Table 2:** Comparison of different methods in the analysis of APE data.

	Logarithmic APE concentration (mean ± SE)			Prediction for having any symptom [with 1-unit increase in log(APE)]	
Method	Period 3	Period 4	*p*-Value	OR (95% CI)	*p*-Value
MI	−0.48 ± 0.23	−3.09 ± 0.26	< 0.0001	1.07 (1.00–1.14)	0.047
Impute log(LOD)/2	−0.019 ± 0.11	−0.92 ± 0.12	< 0.0001	1.13 (0.99–1.28)	0.075
Impute log(LOD)	−0.28 ± 0.15	−1.80 ± 0.16	< 0.0001	1.10 (1.00–1.21)	0.062
Exclude nondetects	0.29 ± 0.12	−0.12 ± 0.16	0.020	1.12 (0.95–1.32)	0.17

Abbreviations: CI, confidence interval; OR, odds ratio.
